# Acute Liver Failure in a COVID-19 Patient Without any Preexisting Liver Disease

**DOI:** 10.7759/cureus.10045

**Published:** 2020-08-26

**Authors:** Dhineshreddy Gurala, Hassan Al Moussawi, Jobin Philipose, Jeffrey R Abergel

**Affiliations:** 1 Internal Medicine, Northwell Health-Staten Island University Hospital, Staten Island, USA; 2 Gastroenterology and Hepatology, Northwell Health-Staten Island University Hospital, Staten Island, USA

**Keywords:** covid 19, acute liver failure, cytokine release syndrome

## Abstract

In December 2019, an outbreak of novel coronavirus started in Wuhan, China, which gradually spread to the entire world. The World Health Organization (WHO) on February 11, 2020, officially announced the name for the disease as coronavirus disease 2019, abbreviated as COVID-19. It is caused by severe respiratory distress syndrome coronavirus 2 (SARS-CoV-2). The WHO declared SARS-CoV-2 as a pandemic on March 11, 2020. SARS-CoV-2 mainly causes fever as well as respiratory symptoms such as cough and shortness of breath. Gastrointestinal/hepatic sequelae such as diarrhea, nausea, vomiting, and elevated liver enzymes have been reported as well. Studies and data so far on coronavirus infections from China, Singapore, and other countries showed that liver enzymes elevation could be seen in 20-50% of cases. More severe disease can correlate with the worsening of liver enzymes. However, acute liver failure in patients with COVID-19 has not been described. Herein we report a case of acute liver failure in an elderly patient with COVID-19 infection who did not have a history of preexisting liver disease.

## Introduction

Until recently, seven types of coronaviruses had been reported to cause infections in humans [1]. Coronaviruses can use animal hosts and then can evolve to infect humans. This process is thought to explain the emergence of SARS-CoV (severe respiratory distress syndrome coronavirus) in 2003, MERS-CoV (Middle Eastern Respiratory Syndrome) in 2012, and SARS-CoV-2 in 2019. SARS-CoV-2 has 82% genome sequence similarity to SARS-CoV and 50% genome sequence homology to MERS-CoV. COVID-19 symptoms range from mild (fever, cough, or dyspnea) to moderate (respiratory failure requiring oxygen support) and can progress to ARDS (acute respiratory distress syndrome) and multiorgan failure. In one of the earlier studies, 80% of cases were mild, but the mortality rate ranged from 1.86% to 9.86% [2]. Higher mortality rates were reported in countries like Italy, possibly secondary to resource depletion in an overwhelmed health care system. Gastrointestinal symptoms such as diarrhea have been reported in approximately 2-10% of patients [3], with a lower rate in China (3.7%) as compared to Singapore (17%) [4]. Liver injury has been reported in 60% of patients with SARS-CoV [5] and has also been reported in patients infected with MERS-CoV [6]. Studies also suggest that SARS-CoV-2 can affect the liver [7-9]. In a recent study published in Shanghai, 75 (50.7%) out of 148 patients were found to have elevated liver enzymes with SARS-CoV-2 [7]. In another study published in the Lancet in February 2020 by Huang et al., an increase in aspartate aminotransferase (AST) was observed in 62% in intensive care unit (ICU) patients compared to 25% in non-ICU patients, indicating that more severe disease correlates with worsening of liver enzymes [10]. Several other studies showed that liver injury in the form of an increase in AST/alanine aminotransferase (ALT) levels with a mild increase in bilirubin ranging from 14.8% to 53% [11]. In patients who died of SARS-CoV-2, liver injury was reported as high as 58.06% [12]. The highest levels recorded included an ALT of 7,590 U/L and an AST of 1,445 U/L [13]. Generally speaking, transaminase elevations are mild in patients with COVID-19. Here, we report a case of acute liver failure in an elderly patient with COVID-19 infection who did not have a history of preexisting liver disease.

## Case presentation

An 80-year-old male with a medical history of diabetes, hypertension, dyslipidemia, asthma, coronary artery disease with bypass graft, atrial fibrillation on warfarin, and heart failure with preserved ejection fraction with an automatic implantable cardiac defibrillator and pacemaker presented to the emergency department (ED) in March 2020 with intermittent fever, productive cough, and shortness of breath (SOB) for four to five days. He initially started noticing fever that was partially relieved by acetaminophen five days prior to presentation (maximum temperature of 102°F at home). This was associated with SOB on exertion, which progressed to SOB at rest and a productive cough. He denied any recent travel, contact with sick person, herbal medications use, or a recent change in home medications. His home medications included oral warfarin daily, oral metoprolol tartrate two times daily, oral metformin ER daily, oral aspirin daily, oral atorvastatin, and budesonide-formoterol inhaler twice daily. The review of systems was otherwise negative. The patient did not have a history of smoking, alcohol consumption, illicit drugs, or high-risk sexual behavior. Vitals at the time of admission showed a temperature of 101.6°F, heart rate of 80 beats/minute, blood pressure 140/70 of mm Hg, respiratory rate of 20 breaths/minute, and oxygen saturation of 98% on room air. Physical examination was positive for bilateral wheeze and rhonchi in all lung fields, 1+ pedal edema bilateral. His chest was without spider angiomas and abdomen with no hepatosplenomegaly, and he had no shifting dullness, with normoactive bowel sounds and no palmar erythema. On neurological examination, he was alert, oriented to time, place/person, followed commands, and had no focal deficits. Laboratory examination results are shown in Table 1.

**Table 1:** Laboratory results WBC, white blood cell; RBC, red blood cell; eGFR, estimated glomerular filtration rate

Laboratory results	Day 0
WBC count	2.20 (normal: 4.8-10.8 K/uL)
RBC count	2.75 (normal: 4.7-6.1 M/uL)
Hemoglobin	10.4 (normal: 14-18 g/dL)
Hematocrit	30.2 (normal: 42-52%)
Platelet count	74 (normal: 13-400 K/uL)
Lymphocyte	1.03 (normal: 1.2-3.4 K/uL)
Serum total protein	6.3 (normal: 6.0-8.0 mg/dL)
Serum albumin	3.7 (normal: 3.5-5.2 mg/dL)
Serum total bilirubin	0.5 (normal: 0.2-1.2 mg/dL)
Serum alkaline phosphatase	78 (normal: 30-115 U/L)
Aspartate aminotransferase	34 (normal: 0-41 U/L)
Alanine aminotransferase	14 (normal: 0-41 U/L)
eGFR	52 (normal: ≥60 mL/min/1.73 m^2^)
Creatinine kinase	754 (normal: 0-225 U/l)

**Figure 1:**
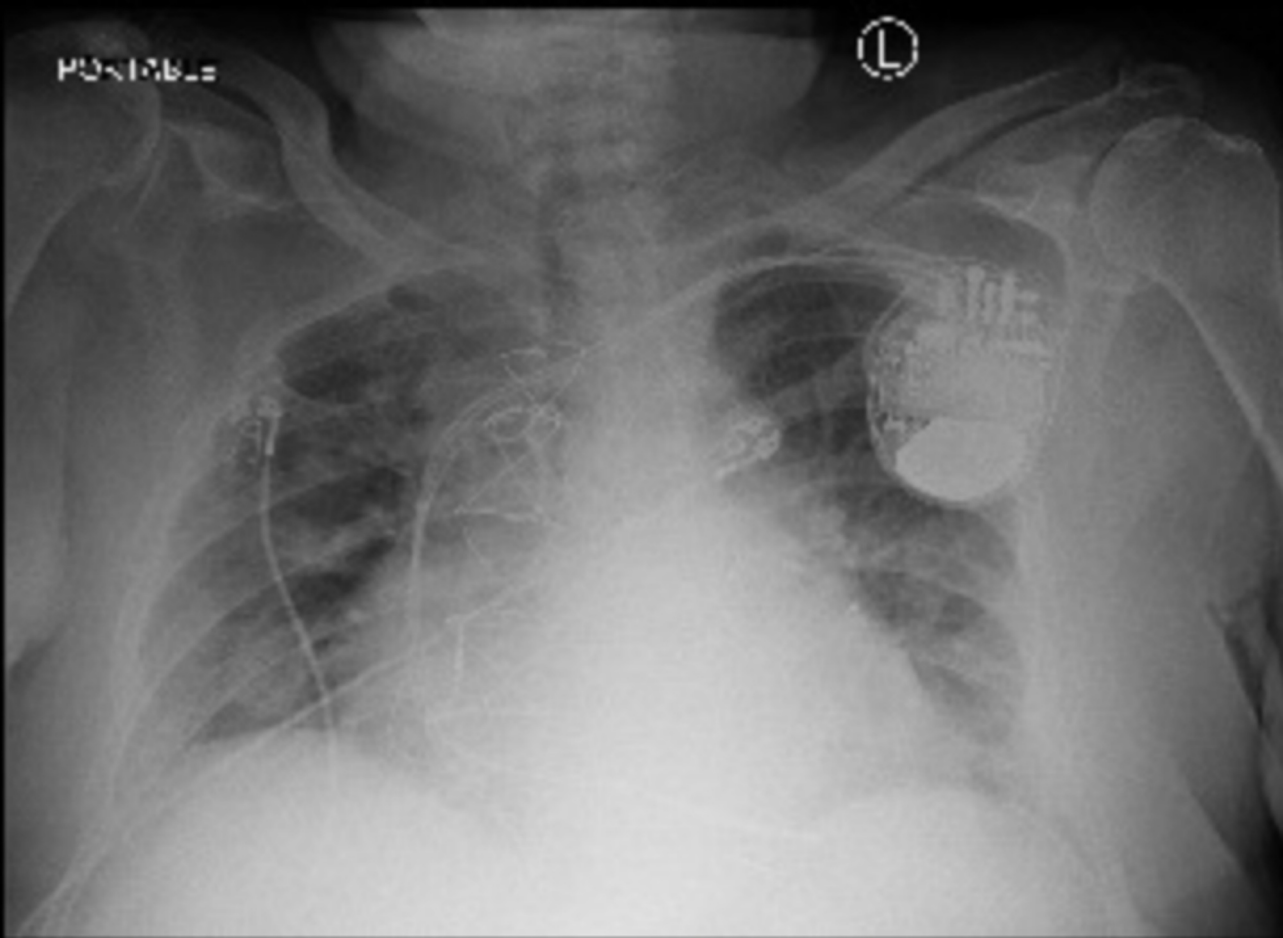
Day 1: portable chest X-ray showing bilateral interstitial infiltrates

**Figure 2:**
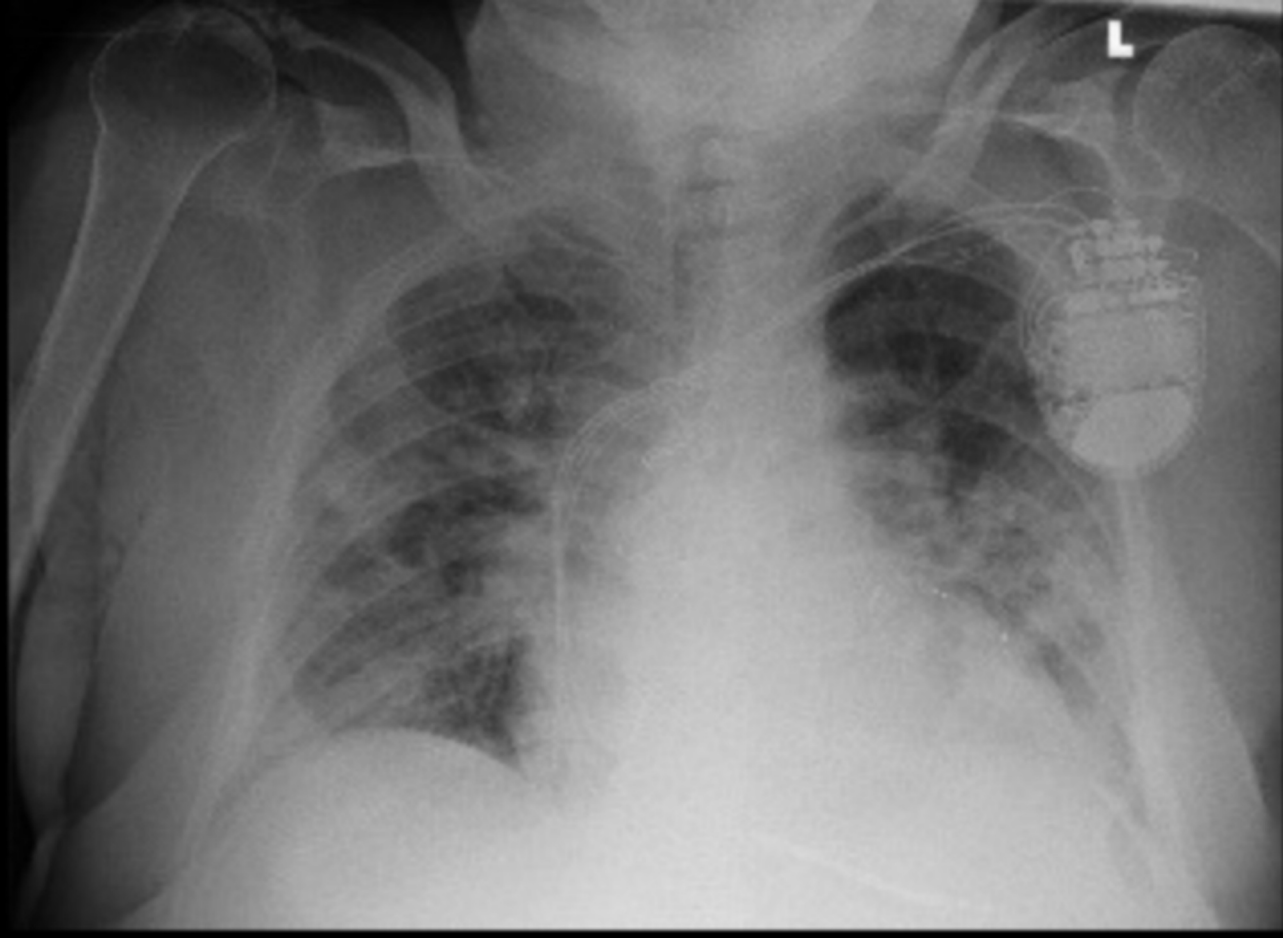
Day 4: portable chest X-ray showing worsening bilateral interstitial infiltrates

**Figure 3:**
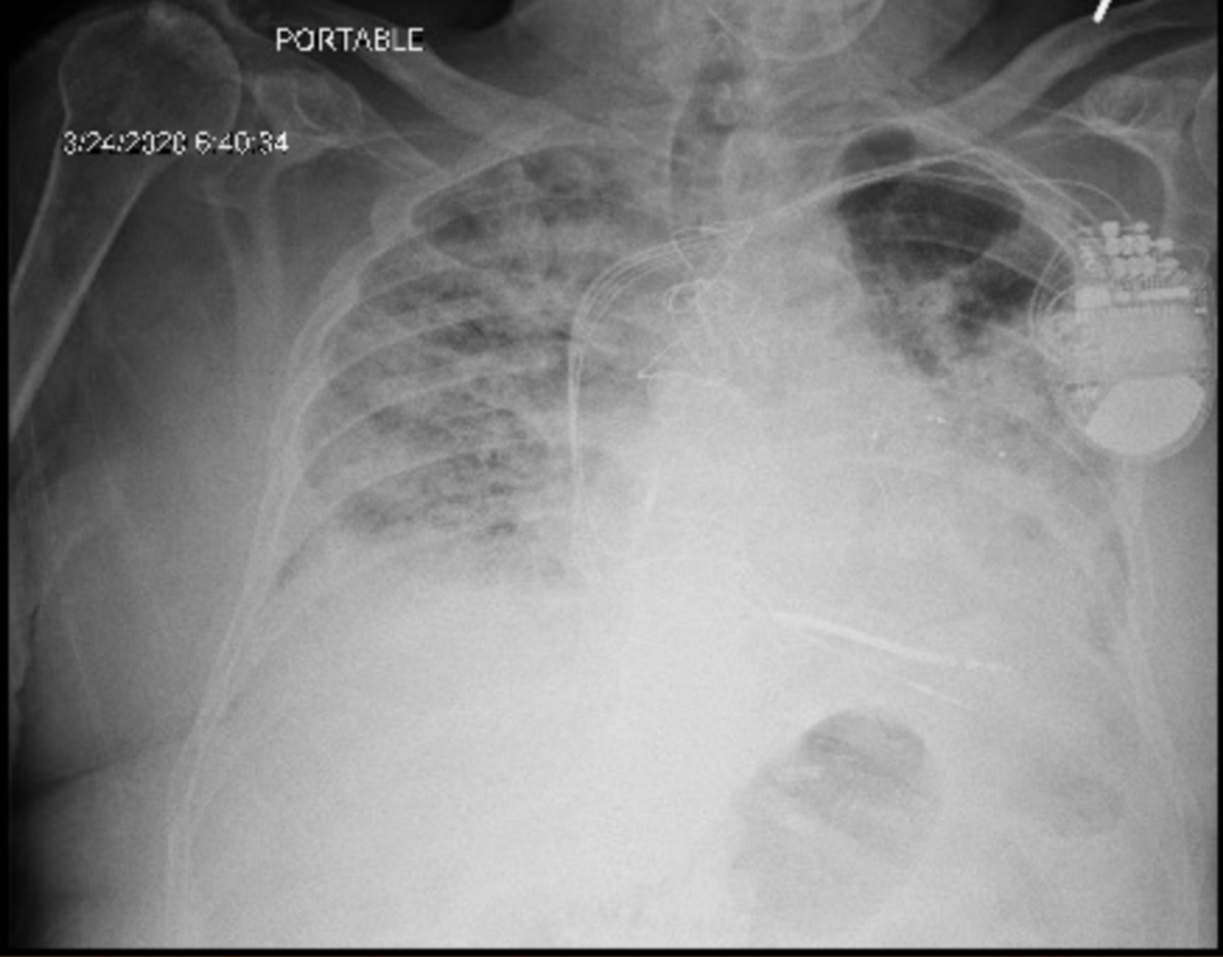
Day 7: progressive worsening bilateral infiltrates

The patient had normal liver enzymes at presentation but had elevated transaminases on day 4. The examination at that time was negative for asterixis or encephalopathy. Atorvastatin was stopped, and the recommendation was made to start N acetylcysteine (NAC), and workup for acute and chronic liver disease was ordered. His respiratory status continued to deteriorate, requiring increased oxygen support. His radiologic findings worsened with enlarging infiltrates on a chest X-ray on day 4, as shown in Figures 1-3.

On day 5, the patient changed his status to do not resuscitate or intubate after the goals of care conversation. He also refused further interventions, including ultrasound abdomen, liver biopsy, or NAC. Progression of the patient’s vital signs and laboratory results are shown in Tables 2, 3.

**Table 2:** Vital signs BP, blood pressure; HR, heart rate; RR, respiratory rate; RA, room air; NC, NC, nasal cannula; NRB, non-rebreather mask

Vital signs	Day 0	Day 1	Day 2	Day 3	Day 4	Day 5	Day 6	Day 7	Day 8
Temperature (°F)	101.6	97.8	103.1	102.1	99.7	96.7	98.7	96.2	93.5
BP (mm Hg)	140/70	150/68	138/62	144/62	152/72	140/93	141/93	142/82	99/55
HR (beats/min)	80	69	68	62	86	64	64	60	63
RR (per min)	20	18	18	22	22	26	28	22	26
Saturation (%)	98% RA	97% RA	97% RA	97% 2 liters NC	93% 5 liters NC	93% on the venti mask 50%	97% on NRB	90% on NRB	88% on NRB

**Table 3:** Laboratory results during subsequent hospitalization WBC, white blood cell; RBC, red blood cell; eGFR, estimated glomerular filtration rate; INR, international normalized ratio

Laboratory results	Day 0	Day 1	Day 2	Day 3	Day 4	Day 5	Day 6	Day 7	Day 8
WBC count (4.8-10.8 K/uL)	2.20	1.27	5.80	3.85	5.35	8.28	--	10.99	16.27
RBC count (4.7-6.1 M/uL)	2.75	2.38	2.98	2.67	2.60	2.61	--	2.95	3.15
Hemoglobin (14-18 g/dL)	10.4	9.0	10.7	9.9	9.6	9.6	--	10.8	10.9
Hematocrit (42-52%)	30.2	27.8	32.3	28.8	28.3	28.9	--	31.5	33.1
Platelet count (13-400 K/uL)	74	55	90	92	83	49	--	76	66
Lymphocyte (1.2-3.4 K/uL)	1.03	0.93	1.65	1.49	1.36	1.22	--	0.63	1.02
Serum total protein (6.0-8.0 mg/dL)	6.3	6.4	6.7	6.2	6.2	6.3	--	6.4	6.6
Serum albumin (3.5-5.2 mg/dL)	3.7	3.6	3.8	3.4	3.4	3.4	--	3.3	3.3
Serum total bilirubin (0.2-1.2 mg/dL)	0.5	0.3	0.4	0.6	1.2	3.4	--	5.2	8.4
Serum alkaline phosphatase (30-115 U/L)	78	71	70	107	145	322	--	357	358
Aspartate aminotransferase (0-41 U/L)	34	27	43	95	3223	>7000	--	2619	1361
Alanine aminotransferase (0-41 U/L)	14	13	14	37	1553	3737	--	2588	1989
Gamma glutamyltransferase (0-41 U/L)									158
eGFR (≥60 mL/min/1.73 m^2^)	52	63	52	52	47		--	19	11
INR		2.54	1.88	1.59	1.87	2.66	--	5.05	8.94
Fibrinogen (mg/dL) (204-570)		>700							
Procalcitonin (0.02-0.10 ng/mL)		0.88		1.16					3.07
Ferritin (30-400 ng/mL)		436							

On day 6, he was disoriented to time and place, his physical examination was positive for asterixis, and international normalized ratio (INR) began to rise on day 7 and day 8 despite the fact that coumadin was held on day 1. The patient was hypertensive until day 8 when his blood pressure dropped to 99/55 mm Hg. The clinical picture was suggestive of acute liver failure. Transfer to live transplant center was not attempted per the family’s request. Tylenol level was <5 (normal: 10-30 ug/mL); other biochemical tests for acute and chronic liver disease are shown in Table 4.

**Table 4:** Transaminitis workup Ig, immunoglobulin; Ag, Ag; AB, antibody; HSV herpes simplex virus; EBV, Epstein-Barr virus; CMV, cytomegalovirus; PCR, polymerase chain reaction; HIV human immunodeficiency virus; ANA, antinuclear antibodies; AMA, antimitochondrial antibodies; ASMA, antismooth muscle antibodies; LKM, liver kidney microsomal; TIBC, total iron-binding capacity

Viral panel	
Hepatitis A IgM antibody	Non-reactive
Hepatitis B surface Ag	Non-reactive
Hepatitis B surface Ab	Non-reactive
Hepatitis B core IgM Ab	Non-reactive
Hepatitis C Ab	Non-reactive
HSV-1 IgM Ab	Non-reactive
HSV-1 IgG Ab	Positive
HSV-2 IgM Ab	Non-reactive
HSV-2 IgG Ab	Non-reactive
EBV IgM Ab	Non-reactive
CMV PCR	Non-reactive
HIV PCR	Non-reactive

Autoimmune and metabolic panel	
ANA	<1:20
AMA	<1:20
ASMA	<1:20
Anti-LKM 1 Ab	<20 (normal: 0-20 units)
IgA	442 (normal: 84-499 mg/dL)
IgM	106 (normal: 35-242 mg/dL)
IgG	1148 (normal: 610-1160 mg/dL)
Serum iron	236 (normal: 35-150 ug/dL)
TIBC	253 (normal: 220-430 ug/dL)
Ferritin	436 (normal: 30-400 ng/mL)
Ceruloplasmin	36 (normal: 15-30 mg/dL)

The patient then developed cytokine release syndrome (CRS) (elevated interleukin [IL]-6 and IL-10 as mentioned in Table 5), and he expired on day 9.

**Table 5:** Cytokine markers IL, interleukin; IFN, interferon

Cytokine markers	
IL-6	59 (normal: 0-15.5 pg/mL)
IL-10	18.1 (normal: <2 pg/mL)
IL-2	<31 (normal: 0-31.2 pg/mL)
IL-17	<5 (normal: <13 pg/mL)
IFN gamma	23 (normal: <5 pg/mL)

## Discussion

COVID-19 is a pandemic illness that primarily affects the respiratory system with a wide spectrum of disease presentation that ranges from mild disease (fever, cough) to severe (ARDS, multiorgan failure). The gastrointestinal/hepatic systems are the next most commonly affected, with symptoms such as nausea, vomiting, diarrhea, and an increase in liver enzymes. Currently, studies on the exact pathophysiology of liver injury in these patients are limited, but it is believed either to be a direct effect of the virus or immune-mediated inflammatory response, such as CRS, hypoxemia, and failure of innate immune regulation, or to be drug-induced.

1) It is postulated that both SARS-CoV-2 and SARS-CoV bind to angiotensin-converting enzyme 2 (ACE2) receptors to enter the target cell [14] where the virus replication begins and starts to infect cells of the upper respiratory tract. Based on the scRNA-seq data, Chai et al. [15] found that ACE 2 receptors also found in the hepatobiliary system (high in bile duct cells, cholangiocytes, when compared to liver cells). Cholangiocytes play a critical role in liver regeneration and immune responses [16]. Thus, the authors concluded that potential damage of cholangiocytes by 2019-nCoV might lead to profound consequences in the liver rather than the direct effect of the virus on hepatocytes.

2) CRS is a group of disorders caused by a wide variety of inflammatory etiologies, resulting in a profound increase in inflammatory markers such as IL-2, IL-7, IL-6 granulocyte colony-stimulating factor, interferon-γ inducible protein 10, monocyte chemo-attractant protein 1, macrophage, inflammatory protein 1-α, and tumor necrosis factor-α. This can ultimately lead to hemodynamic instability, multiorgan dysfunction, and death [17]. Elevations in IL-6, IL-10, procalcitonin, and ferritin, as well as thrombocytopenia have been associated with severe COVID and potentially severe liver injury as seen in our patient [18].

3) Ischemic hepatitis, also known as shock liver, is characterized by a significant increase in serum aminotransferases due to reduced oxygen delivery to the liver, usually seen in shock and thromboembolic disease [19].

4) Clinicians should also consider drug-induced liver injury due to hepatotoxicity associated with drugs used in treating COVID such as lopinavir, ritonavir, and hydroxychloroquine that are recently approved by the FDA for the treatment of COVID.

Our patient, who had no previous history of liver disease and normal liver enzymes at presentation, developed elevated liver enzyme levels on day 4. Initial differential diagnosis was broad, including ischemic hepatitis, drug-induced liver injury, viral hepatitis, cholestasis of sepsis, and autoimmune diseases. On laboratory workup, viral infections such as hepatitis (A, B, C), Epstein-Barr virus, cytomegalovirus, herpes simplex virus, HIV, autoimmune, and metabolic causes were ruled out. Since his blood pressure was stable until day 8 of his hospitalization without any pressor support, ischemic hepatitis was unlikely [20]. Tylenol toxicity was excluded (levels were less than 5 ug/mL).

The remainder of the patient’s medications were reviewed, and none of the patient’s medications was likely to be the culprit. For example, the patient’s home medication coumadin is a rare cause of acute liver injury and usually results in a cholestatic pattern rather than a hepatocellular one, which is what our patient demonstrated. Aspirin has been associated with an increase in liver enzymes but usually only with dosages of more than 1,800 to 3,200 mg daily. Metoprolol and metformin have been associated with only mild elevations in liver enzymes. Hydroxychloroquine used to treat SARS-CoV-2 has been rarely associated with clinically apparent liver injury. A single case series (two cases) of acute liver failure attributed to hydroxychloroquine was published, but these patients took the medication for more than two weeks.

## Conclusions

In summary, we describe the first case of acute liver failure caused by the COVID-19 infection. Acute liver failure was diagnosed clinically by rising liver function tests and INR, as well as progressive encephalopathy. We could not conclusively prove that the COVID-19 was the etiologic agent as the patient declined a liver biopsy. However, alternative causes of acute liver failure were effectively ruled out. Bloodwork did not identify another etiology, and the patient’s hypotension was too late in his course and too mild to cause ischemic hepatopathy. Additionally, none of his medications was among the usual suspect for acute liver failure. As we learn more about this new infection, we expect to better understand the spectrum, pathophysiology, and treatment of the resultant liver injury.

## References

[1] Zheng J (2020). SARS-CoV- 2: an emerging coronavirus that causes a global threat. Int J Biol Sci.

[2] Guan WJ, Ni ZY, Hu Y (2020). Clinical characteristics of 2019 novel coronavirus infection in China. N Engl J Med.

[3] Yeo C, Kaushal S, Yeo D (2020). Enteric involvement of coronaviruses: is faecal-oral transmission of SARS-CoV-2 possible?. Lancet Gastroenterol Hepatol.

[4] Young BE, Ong SWX, Kalimuddin S (2020). Epidemiologic features and clinical course of patients infected with SARS-CoV-2 in Singapore. JAMA.

[5] Chau TN, Lee KC, Yao H (2004). SARS-associated viral hepatitis caused by a novel coronavirus: report of three cases. Hepatology.

[6] Alsaad KO, Hajeer AH, Al Balwi M (2018). Histopathology of Middle East respiratory syndrome coronovirus (MERS-CoV) infection - clinicopathological and ultrastructural study. Histopathology.

[7] Fan Z, Chen L, Li J (2020). Clinical features of COVID-19-related liver damage. Clin Gastroenterol Hepatol.

[8] Chen N, Zhou M, Dong X (2020). Epidemiological and clinical characteristics of 99 cases of 2019 novel coronavirus pneumonia in Wuhan, China: a descriptive study. Lancet.

[9] Zhang C, Shi L, Wang FS (2020). Liver injury in COVID- 19: management and challenges. Lancet Gastroenterol Hepatol.

[10] Huang C, Wang Y, Li X (2020). Clinical features of patients infected with 2019 novel coronavirus in Wuhan, China. Lancet.

[11] Xu L, Liu J, Lu M, Yang D, Zheng X (2020). Liver injury during highly pathogenic human coronavirus infections. Liver Int.

[12] Huang Y, Zhou H, Yang R (2020). Clinical characteristics of 36 non-survivors with COVID-19 in Wuhan, China [PREPRINT]. medRxiv.

[13] Chen N, Zhou M, Dong X (2020). Epidemiological and clinical characteristics of 99 cases of 2019 novel coronavirus pneumonia in Wuhan, China: a descriptive study. Lancet.

[14] Hoffmann M, Kleine-Weber H, Krüger N, Müller M, Drosten C, Pöhlmann S (2020). The novel coronavirus 2019 (2019-nCoV) uses the SARS-1 coronavirus receptor2 ACE2 and the cellular protease TMPRSS2 for entry into target cells [PREPRINT].

[15] Chai X, Hu L, Zhang Y (2020). Specific ACE2 expression in cholangiocytes may cause liver damage after 2019-nCoV infection [PREPRINT]. bioRxiv.

[16] Banales JM, Huebert RC, Karlsen T, Strazzabosco M, LaRusso NF, Gores GJ (2019). Cholangiocyte pathobiology. Nat Rev Gastroenterol Hepatol.

[17] Zhang W, Zhao Y, Zhang F (2020). The use of anti-inflammatory drugs in the treatment of people with severe coronavirus disease 2019 (COVID- 19): the perspectives of clinical immunologists from China. Clin Immunol.

[18] Lippi G, Plebani M, Henry BM (2020). Thrombocytopenia is associated with severe coronavirus disease 2019 (COVID-19) infections: a meta-analysis. Clin Chim Acta.

[19] Waseem N, Chen PH (2016). Hypoxic hepatitis: a review and clinical update. J Clin Transl Hepatol.

[20] Khan H, Phillipose J, Ahmed M, Deeb L (2018). Athlete's hepatitis in a young healthy marathon runner. Case Rep Gastroenterol.

